# The *MNK-SYNGAP1* axis in specific learning disorder: gene expression pattern and new perspectives

**DOI:** 10.1007/s00431-025-06089-6

**Published:** 2025-03-19

**Authors:** Cansu Mercan Isik, Elif Burcu Tuzemen Bayyurt, Nil Ozbilum Sahin

**Affiliations:** 1 Department of Child and Adolescent Psychiatry, Faculty of Medicine, Cumhuriyet University, Sivas, Turkey; 2 Department of Medical Biology, Faculty of Medicine, Cumhuriyet University, 58140 Sivas, Turkey; 3 Department of Molecular Biology and Genetic, Faculty of Science, Cumhuriyet University, 58140 Sivas, Turkey

**Keywords:** SLD, MNK1, SYNGAP1, Gene expression, QPCR

## Abstract

Specific learning disorder (SLD) is a neurodevelopmental disorder that significantly affects children’s academic performance. This study aimed to investigate the expression levels of the MAP Kinase Interacting Serine/Threonine Kinase 1–2 (*MNK1*, *MNK2*), Synaptic Ras GTPase Activating Protein 1 (*SYNGAP1*) genes, and the long non-coding RNA Synaptic Ras GTPase Activating Protein 1-Anti Sense1 (*SYNGAP1-AS1*), which are believed to play a key role in neurodevelopmental pathways, in children with SLD. Understanding the role of these genes in synaptic plasticity and cognitive function may provide insights into the molecular mechanisms underlying SLD. This study included 38 children diagnosed with SLD and 35 healthy controls aged 6 to 16. RNA was isolated from blood samples, and gene expression levels were measured using quantitative polymerase chain reaction (qPCR). The statistical analysis was conducted to compare the expression levels between the SLD and control groups and within SLD subgroups based on severity and sex. *MNK1* and *SYNGAP1* expression levels were significantly upregulated in the SLD group compared to the control group (8.33-fold and 16.52-fold increase, respectively; *p* < 0.001). *lncSYNGAP1-AS1* showed a 26.58-fold increase, while *MNK2* was downregulated by 2.2-fold, although these changes were not statistically significant. No significant differences were observed between sexes or between the severity subgroups of SLD.

*Conclusion*: he upregulation of *MNK1* and *SYNGAP1* in children with SLD suggests their involvement in the neurodevelopmental pathways associated with cognitive processes such as learning and memory. These findings provide a foundation for future research into the molecular basis and potential therapeutic targets of SLD.

**Table:** 

What is known: • *SYNGAP1 is a key regulator of synaptic plasticity and learning, primarily functioning through Ras signaling inhibition. Its deficiency impairs long-term potentiation (LTP) and is associated with neurodevelopmental disorders (NDDs) such as autism spectrum disorder (ASD) and intellectual disability.* • *The MAPK/ERK pathway plays a crucial role in learning and memory, and its dysregulation has been linked to several neurological conditions. MNK1/2 interacts with SYNGAP1 in synaptic signaling.*	
What is new: • *This study is the first to demonstrate significant upregulation of SYNGAP1 and MKNK1 in children with SLD.* • *Understanding the role of the MKNK-SYNGAP1 axis may guide the development of targeted therapies aimed at enhancing synaptic plasticity to improve learning and memory outcomes in children with SLD.*

What is known:

• *SYNGAP1 is a key regulator of synaptic plasticity and learning, primarily functioning through Ras signaling inhibition. Its deficiency impairs long-term potentiation (LTP) and is associated with neurodevelopmental disorders (NDDs) such as autism spectrum disorder (ASD) and intellectual disability.*

• *The MAPK/ERK pathway plays a crucial role in learning and memory, and its dysregulation has been linked to several neurological conditions. MNK1/2 interacts with SYNGAP1 in synaptic signaling.*

What is new:

• *This study is the first to demonstrate significant upregulation of SYNGAP1 and MKNK1 in children with SLD.*

• *Understanding the role of the MKNK-SYNGAP1 axis may guide the development of targeted therapies aimed at enhancing synaptic plasticity to improve learning and memory outcomes in children with SLD.*

## Introduction

Neurodevelopmental disorders (NDDs) are a diverse group of psychiatric conditions marked by early-onset abnormalities in brain and central nervous system development. These include intellectual disability, attention deficit/hyperactivity disorder, specific learning disorder (SLD), and autism spectrum disorder [1]. SLD is characterized by significant difficulties in learning and using academic skills, such as reading, writing, and math, which are below expectations for a person’s age and education. According to the Diagnostic and Statistical Manual of Mental Disorders-5 (DSM-5), SLD affects 5–15% of school-aged children and 4% of adults. While the exact causes of SLD remain unclear, genetic factors, central nervous system dysfunctions, and information processing issues (e.g., input, integration, and memory problems) are considered key contributors [2].

Synaptic plasticity is a fundamental mechanism for learning and memory formation. The formation of new memories requires both structural and functional remodeling of synapses [3]. The long-term increase or decrease of synaptic strengthening, known as long-term potentiation (LTP) or long-term depression (LTD), forms the cellular basis of memory formation [4]. These processes occur by triggering neuronal activation, modification of molecules, and new protein synthesis through intracellular signaling pathways [5]. Researches have revealed that epigenetic regulation plays a critical role in synaptic plasticity and memory [3, 6–10].

**Fig. 1:**
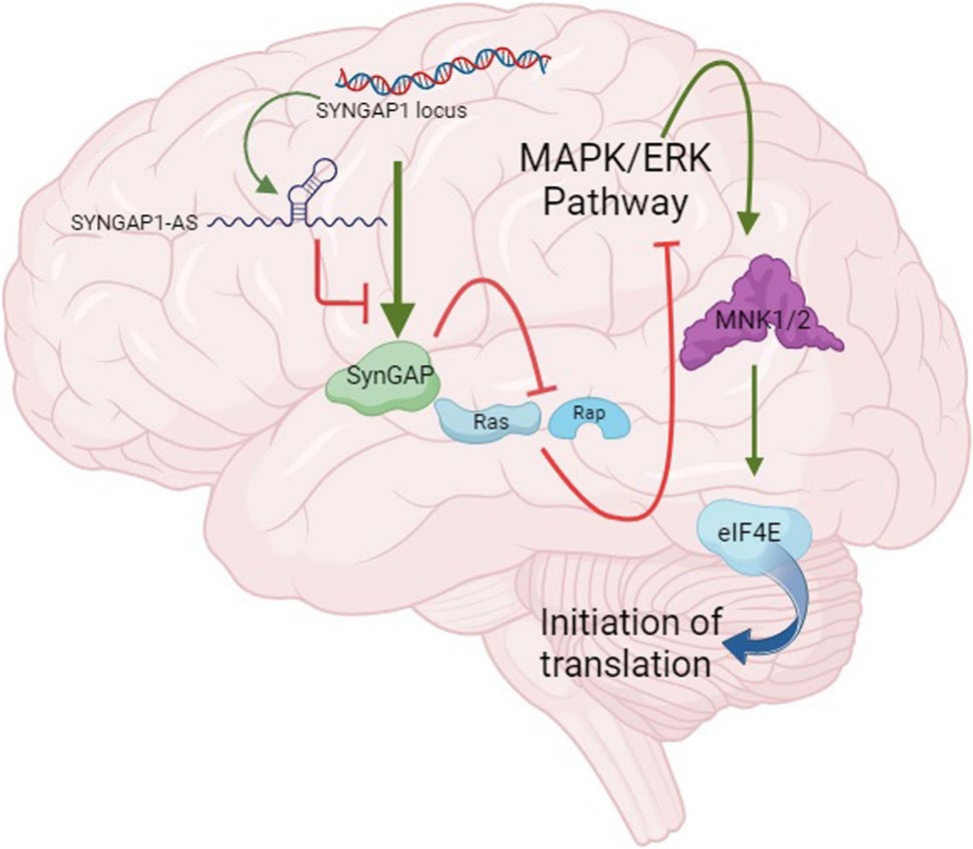
A schematic diagram illustrating the *MKNK-SYNGAP1* Axis and its associated proteins/elements. *SYNGAP* reduces redundant (noise) signals by suppressing Ras-ERK signaling at rest. During synaptic activation (learning), *SYNGAP* is phosphorylated and suppressed, effectively increasing this signal

The Synaptic Ras GTPase-Activating Protein 1 (*SYNGAP-1*) gene, which is particularly important for learning and memory plays a complex role in neurodevelopment and ongoing neurological function [11, 12]. *SYNGAP* encodes a GTPase-activating protein that is selectively expressed in the brain and plays critical roles in neuronal function and brain development by regulating biochemical signaling in neurons. *SYNGAP* is a negative regulator of small GTPases such as Ras and Rap and is required for synaptic development, structure, function, and plasticity. *SYNGAP* is expressed by the frontal cortex and is found at particularly high levels in the hippocampus [11]. Mutations in *SYNGAP1*, which encodes the *SYNGAP* protein, have been identified in patients with intellectual disability, autism spectrum disorder (ASD), severe epilepsy, and schizophrenia [13]. Long non-coding RNAs (lncRNAs) constitute a large and diverse group of non-protein coding RNAs, defined as transcripts consisting of more than 200 nucleotides. LncRNAs regulate gene expression through various mechanisms, including transcriptional interference, chromatin remodeling, interaction with antisense transcripts, generation of small RNAs, binding to specific proteins to modulate their activity, participating RNA–protein complexes, and influencing protein localization within the cell [14]. Specifically, *lncSYNGAP1-AS1*, an antisense lncRNA transcribed from the *SYNGAP1* gene, may regulate *SYNGAP1* expression through epigenetic mechanisms, thereby affecting neuronal function and synaptic plasticity. *MKNK1* encodes serine/threonine kinase 1 (*MNK1*), a key regulator of protein synthesis and synaptic function, which is activated by ERK in the extracellular signal-regulated MAPK pathway. *MNK1* is the predominant *MNK* isoform in the brain [15]. *MNK1* phosphorylates the eukaryotic initiation factor eIF4E and regulates translation initiation, thereby enhancing protein synthesis at synaptic sites to support the long-term synaptic changes required for memory formation [16]. *SYNGAP* reduces redundant (noise) signals by suppressing Ras-ERK signaling at rest. During synaptic activation (learning), *SYNGAP* is phosphorylated and inhibited, leading to an increased signal [17]. The relationship among *SYNGAP1*, *SYNGAP1-AS1*, and *MNK1* is illustrated in Fig. 1.

Changes in gene expression are widely studied to characterize various diseases and are used to understand molecular and cellular processes in complex diseases [18]. In many countries, the number of children diagnosed with neurodevelopmental disorders has been reported to increase by approximately 10,000 cases annually [19]. However, this increase is unlikely to be solely the result of genetic factors, as there is no reason to suspect that mutation rates have increased significantly in recent years. Instead, a more likely explanation is that epigenetic processes contribute to this trend [20]. In addition, molecular evidence is gaining important for objective diagnosis and effective treatment. Gene expression is critical for identifying potential biomarkers for neurodevelopmental disorders and elucidating their etiology [21].

For these reasons, our study aims to examine the expression levels of MNK1, MNK2, and SYNGAP1 genes, which are strongly implicated in neurodevelopmental disorders, as well as SYNGAP1-AS, a related lncRNA, in children with SLD. To the best of our knowledge, there is no such study in the literature.

## Material and methods

### Participants and sample collection

This study was conducted in collaboration with the Departments of Child Psychiatry, Medical Biology, and Molecular Biology and Genetics at Sivas Cumhuriyet University Faculty of Medicine. The initial ethical approval for participant recruitment and blood collection was granted by the Clinical Research Ethics Committee of Sivas Cumhuriyet University on 28 March 2023 (Decision No: 2023-03/10). A subsequent approval from the Non-Interventional Clinical Research Ethics Committee of Sivas Cumhuriyet University on 21 December 2023 (Decision No: 2023-12/53) permitted the use of the same blood samples for the present study.

G*Power (3.1) program was used for power calculation. The sample size was determined by the Biostatistics Department, with a test power calculation of *p* = 0.90718 (*α* = 0.05, *β* = 0.10, and (1-*β*) = 0.90), resulting in the inclusion of 73 participants: 38 children diagnosed with specific learning disorder (SLD) and 35 healthy controls. The severity and diagnosis of specific learning disorder (SLD) were made clinically using DSM-5 diagnostic criteria, taking into account factors such as the degree of impairment in the individual’s academic skills (by applying reading, writing, and math tests), its impact on daily life, and response to intervention [2]. Children with SLD exhibited combined symptoms of dyslexia, dysgraphia, and dyscalculia.

### Inclusion criteria

- Children aged 6–16 diagnosed with pure SLD
- No history of special education
- Matched control group based on age, gender, IQ, and socio-cultural factors

### Exclusion criteria

- Presence of other psychiatric disorders
- Chronic medical conditions, auditory or visual impairments, or current medication use for treatments
- Comorbid Autism Spectrum Disorder (ASD) or ADHD (based on Conners’ Parent Rating Scale-Revised Short Form (CPRS-RS) and DSM-5 criteria)
- Severe intellectual disability, psychosocial deprivation, or inadequate education
- All participants and their legal guardians provided written and verbal informed consent, and the study adhered to the principles of the Declaration of Helsinki.

### Blood sample collection and RNA isolation

Blood samples were collected in RNA Stabilizer Tubes (NucleoGene, NG20200803, Turkey/Istanbul) to preserve RNA integrity at room temperature. Samples were stored at − 20 °C until RNA isolation was performed in the Medical Biology Department. Total RNA was extracted using the Hybrid-R RNA isolation kit (GeneAll, Cat. No.: 305–101, South Korea) following the manufacturer’s protocol.

### Neuropsychological and clinical assessments

A sociodemographic data form was used to collect participant details. Psychiatric evaluations were conducted using the Turkish version of the Schedule for Affective Disorders and Schizophrenia for School-Age Children-Present and Lifetime (K-SADS-PL) [22, 23]. The Wechsler Intelligence Scale for Children-Fourth Edition (WISC-IV) was administered to assess cognitive abilities [24]. Additionally, the revised short form of CPRS-RS was used to rule out ADHD, which could coexist with SLD [25]. Each child completed assessments including reading, writing, and mathematics tests to evaluate these academic skills in detail. Additionally, the clock-drawing and right-left discrimination tests were administered to measure visual perception and hand–eye coordination and to determine the specific SLD subgroup [26].

### Quantitative polymerase chain reaction (qPCR)

Complementary DNA (cDNA) was synthesized using the ABT cDNA synthesis kit (Cat: C03-01–20, Lot: W4F0123-C5, Turkey/Ankara) in a thermal cycler (TECHNE, TC-5000, Maryland/US). RNA concentrations were equalized before synthesis using nuclease-free water. QPCR (LightCycler 96, Roche, Switzerland) was conducted using validated primers for *MNK1*, *MNK2*, *SYNGAP1*, and *SYNGAP1-AS1*, with GAPDH as the endogenous control for normalization. SYBR Green dye (ABT, Cat: Q03-01–05, Lot: W2C0223-Q9, Turkey/Ankara) was used for fluorescence-based detection. Primer sequences are provided in Table 1.

**Table 1:** Primer sequences in the study

Oligo name	Sequence 5′−3′
MKNK1-f	CGAGAGGTGGAGACGCTGTA
MKNK1-r	TGGTTGGTATGGGGGTA
MKNK2-f	TTTTCAGGGTAGGTGGAGATG
MKNK2-r	GGTGGAGTAGGGGAGCAGT
SYNGAP1-f	CTGCCTCCATCTTTCATAGC
SYNGAP1-r	TGGCTGAGACTTGCCCTCTT
SYNGAP1-AS1-f	CTCACCTGCGAATGGATGC
SYNGAP1-AS1-r	AACAAACGCAGCAAATCCTGA

*f* forward,* r* reverse

### Statistical analysis

Statistical analyses were performed using SPSS 23.0. Data normality was assessed with using the Kolmogorov–Smirnov test. Since the data met parametric assumptions, an independent samples *t*-test was used for group comparisons, while the chi-square test was applied for categorical variables. The significance level was set at 0.05. Quantification of gene expression changes was conducted using the 2^ − ΔΔCt method [27]. Data processing was performed using the GeneGlobe Data Analysis Center (https://geneglobe.qiagen.com/us/analyze). Fold change (FC) values for *MNK1*, *MNK2*, *SYNGAP1*, and *lncSYNGAP1-AS1* expression were calculated relative to the control group, normalized to a reference gene. FC > 1 indicates upregulation, FC < 1 indicates downregulation, and FC = 1 shows no change. Statistical significance (*p* < 0.05) was determined using Student’s *t*-test. Figures were generated with GraphPad Prism (version 10.0.0 GraphPad Software, Boston, MA, USA) and BioRender.

## Results

### Clinical characteristics and demographic variables of the participants

A total of 38 children with SLD and 35 controls were included in the study. No significant differences were observed between the groups regarding age, sex, place of residence, family income level, parental education level, maternal pregnancy history, gestational age at birth, mode of delivery, delivery complications, or parental psychiatric history (*p* ≥ 0.05 for all). Data were homogeneously distributed in both groups. The mean clinical characteristics and demographic variables of the participants are presented in Table 2.

**Table 2:** Socio-demographic and clinical characteristics of participants

Variables	Control *N* = 35	SLD *N* = 38	*p*
Sex (*n*, %)			
Male	18	23	0.434
	51.4%	60.53%	
Female	17	15	
	48.6%	39.47%	
Age (mean-years ± *SD*)	10.34** ± **2.6	10.26 ± 2.6	0.892^a^
Severity (*n*, %)			
Severe		21	
		55.3%	
Mild		17	
		44.8%	
Living (*n*, %)			
Province	29	29	0.237
	82.86%	76.32%	
District	6	6	
	17.14%	15.79%	
Village	0	3	
	0.0%	7.89%	
Mental illness in sibling (*n*, %)			
No	32	29	0.082
	91.43%	76.32%	
Yes	3	9	
	8.57%	23.68%	
Family structure (*n*, %)			
Core	31	29	0.306
	88.58%	76.32%	
Large	2	4	
	5.71%	10.53%	
Parents divorced	0	3	
	0.0%	7.89%	
At least one of the parents is deceased	2	2	
	5.71%	5.26%	
Socioeconomic level^*^(*n*, %)			
Low	2	8	0.119
	5.71%	21.05%	
Middle	9	11	
	25.71%	28.95%	
High	24	19	
	68.58%	50%	
Disease during pregnancy (*n*, %)			
No	34	36	0.605
	97.14%	94.74%	
Yes	1	2	
	2.86%	5.26%	
	34	36	
Drug use during pregnancy (*n*, %)			
No	35	36	0.169
	100%	94.74%	
Yes	0	2	
	0.0%	5.26%	
Smoking during pregnancy (*n*, %)			
No	26	33	0.173
	74.29%	86.84%	
Yes	9	5	
	25.71%	13.16%	
Type of birth (*n*, %)			
Normal vaginal birth	28	30	0.911
	80.00%	78.95%	
C/S	7	8	
	20.00%	21.05%	
Time of birth (*n*, %)			
Early	2	8	0.086
	5.71%	21.05%	
Mid	32	27	
	91.43%	71.05%	
Late	1	3	
	2.86%	7.9%	
Birth weight (*n*, %)			
Under 2500 g	2	5	0.501
	5.71%	13.16%	
Between 2500 and 4000 g	31	30	
	88.58%	78.95%	
Over 4000 g	2	3	
	5.71%	7.89%	
Birth complications (*n*, %)			
No	32	32	0.349
	91.43%	84.21%	
Yes	3	6	
	8.57%	15.79%	
	32	32	
Receiving incubator care (*n*, %)			
No	32	29	0.082
	91.43%	76.31%	
Yes	3	9	
	8.57%	23.68%	
Mental illness in mother (*n*, %)			
No	28	32	0.639
	80.00%	84.21%	
Yes	7	6	
	20.00%	15.79%	
Mental illness in father (*n*, %)			
No	30	31	0.634
	85.71%	81.58%	
Yes	5	7	
	14.29%	18.42%	

*SLD* specific learning disorder, *SD* standard deviation

^a^Independent *t*-test, *χ*2 test, and Fisher’s exact test were performed on categorical variables, Statistical significance: *p* ≤ 0.05

^*^The level of income was determined by the minimum wage value on the date of the study for the workers in our country

### Results of qPCR analyses

#### Control vs. SLD

**Fig. 2:**
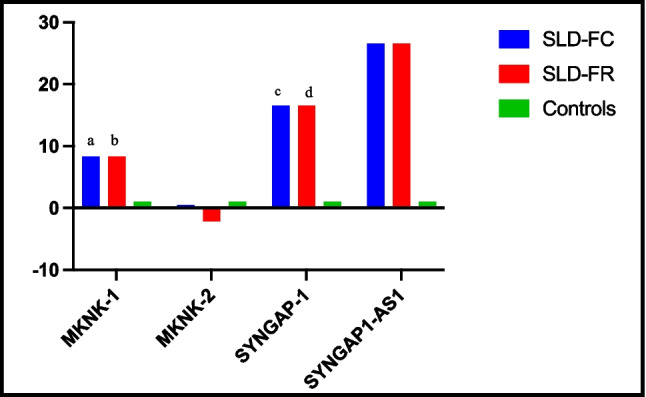
Fold changes and fold regulations resulting from genes expression in groups. SLD, specific learning disorder; FC, fold change; FR, fold regulation. (a, b) *p* ≤ 0.001 compared to controls. (c, d) *p* ≤ 0.05 compared to controls. SLD (*n* = 38), controls (*n* = 35)

Comparison of gene expression levels between children with SLD and controls revealed statistically significant differences for *MNK1* and *SYNGAP1* expression (Fig. 2 and Table 3). *MNK1* expression was 8.33-fold higher in children with SLD compared to controls (*p* < 0.001). *SYNGAP1* expression was 16.52-fold higher in the SLD group (*p* < 0.05). *lncSYNGAP1-AS1* expression was 26.58-fold higher, while *MNK2* expression was 2.2-fold lower in children with SLD compared to controls; however, these differences were not statistically significant.

**Table 3:** Fold changes and fold regulations resulting from genes expression in groups

*Group comparison*	*Gene*	*FC*	*FR*	*p value*
*SLD (n* = *38) vs. control (n* = *35)*				
	*MKNK-1*	8.33	8.33, upregulated	< 0.001^*^
	*MKNK-2*	0.45	− 2.20, downregulated	0.29
	*SYNGAP-1*	16.52	16.52, upregulated	0.02^*^
	*SYNGAP1-AS-1*	26.58	26.58, upregulated	0.207
*Severe (n* = *21) vs. mild (n* = *17)*				
	*MKNK-1*	1.95	1.95, upregulated	0.22
	*MKNK-2*	1.22	1.22, upregulated	0.46
	*SYNGAP-1*	1.58	1.58, upregulated	0.49
	*SYNGAP1-AS-1*	2.42	2.42, upregulated	0.52
*Male (n* = *23) vs. female (n* = *15)*				
	*MKNK-1*	0.58	− 1.74, downregulated	0.06
	*MKNK-2*	1.60	1.60, upregulated	0.76
	*SYNGAP-1*	0.29	− 3.40, downregulated	0.86
	*SYNGAP1-AS-1*	1.04	1.04, upregulated	0.27

*p* value was calculated according to Student’s t-test for each gene examined in comparison of all groups.*p ≤ 0.05

*SLD* specific learning disorder, *FC* fold change, *FR* fold regulation

#### Non-severe vs. mild

**Fig. 3:**
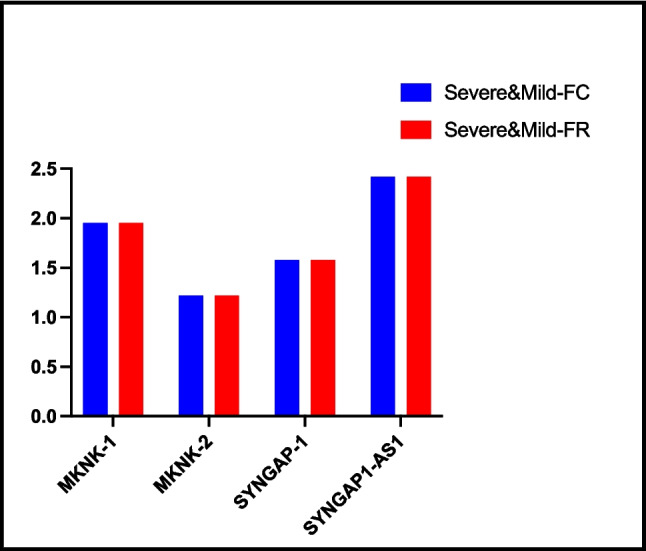
Comparison of fold change and fold regulation values between disease severity in the SLD group. The columns show the increases and decreases in the severe disease group compared to the mild disease group. There was no statistical difference between genders in FC and FR values of genes. FC, fold change; FR, fold regulation. *p* ≤ 0.05. Severe (*n* = 21), mild (*n* = 17)

When we compared the severe and mild groups of children with SLD according to disease severity, there was no significant difference between the groups in *MNK-1*, *MNK-2*, *SYNGAP-1*, and *lncSYNGAP1-AS1* gene expression (Fig. 3 and Table 3).

#### Male vs. female

**Fig. 4:**
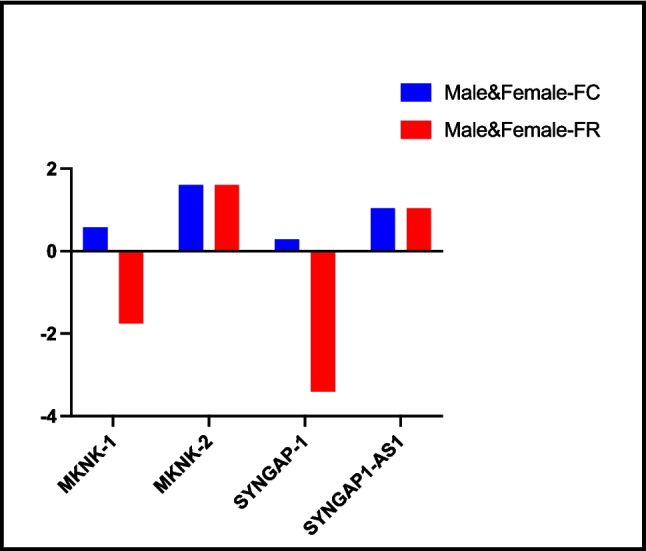
Comparison of fold change and fold regulation values between genders in the SLD group. The columns show the increases and decreases in male gender compared to female gender. There was no statistical difference between genders in FC and FR values of genes. FC, fold change; FR, fold regulation. *p* ≤ 0.05. Male (*n* = 23), female (*n* = 17)

When we evaluated the gene expression of these genes according to gender in the SLD group, there was no significant difference between *MKNK-1*, *MKNK-2*, *SYNGAP-1*, and *lncSYNGAP1-AS1* gene expression (Fig. 4 and Table 3).

### Heatmap with hierarchical clustering, representing the expression levels of genes

**Fig. 5:**
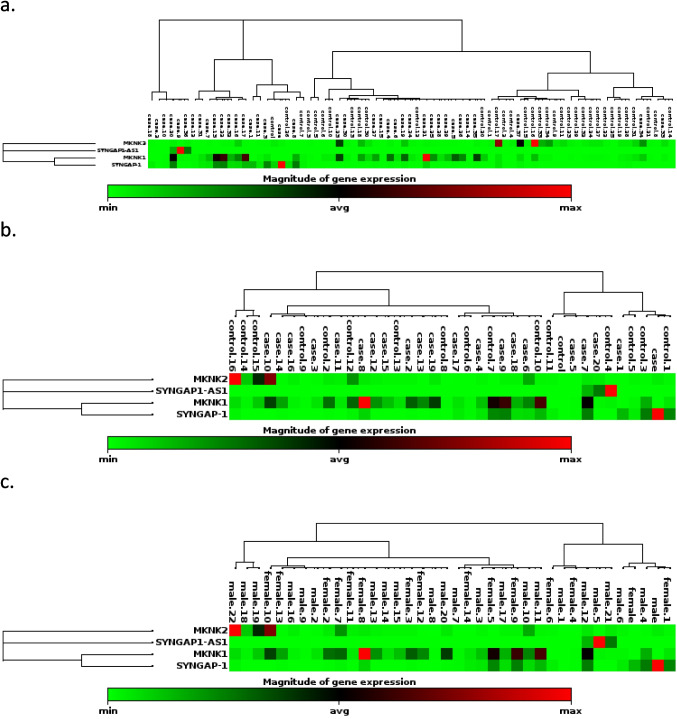
**a** Heatmap showing the expression levels of MKNK1, MKNK2, SYNGAP1, and SYNGAP1-AS1 genes in samples from control and SLD children. **b** Heatmap showing the expression levels of genes across the severity of SLD. **c** Heatmap showing the expression levels of genes across sexes in the SLD group. The color scale, ranging from green to red, reflects the gene expression levels from low to high

A heatmap with hierarchical clustering was generated to visualize the expression patterns of *MNK1*, *MNK2*, *SYNGAP1*, and *lncSYNGAP1-AS1* in control and SLD samples (Fig. 5). The color gradient represents relative gene expression levels: Green indicates low expression, black represents average expression, and red denotes high expression. Genes that are upregulated appear in red, while downregulated genes are shown in green, relative to the average baseline. Children with SLD exhibited upregulated expression of *MNK1*, *SYNGAP1*, and l*ncSYNGAP1-AS1* compared to controls, whereas *MNK2* expression was lower.

## Discussion

Our results in our study investigating the expression of *SYNGAP1*, *lncSYNGAP1-AS1*, and *MNK1-2* in children with SLD are in line with recent discoveries regarding the role of these genes in cognitive and synaptic plasticity pathways. The upregulation of these genes in our study may provide a foundation for investigating their influence neurodevelopmental disorders, suggesting an important link between learning disabilities.

LTP is recognized as one of the fundamental cellular mechanisms involved in learning and memory. Studies on the MAPK pathway and learning have been conducted for many years [28–30]. These findings emphasize the interconnected roles of ERK activation, LTP, and learning and memory processes. Furthermore, MAPK/ERK pathway dysfunction has been associated with many neurological pathologies, including ASD [31–33]. In a study conducted in patients with ASD in 2019, increased activity of MAPK pathways, which are key regulators of synaptogenesis and protein synthesis, was determined [34]. In particular, it was suggested that *p*-MNK1 expression could distinguish patients according to their clinical diagnoses and could constitute a molecular signature of clinical severity in autism spectrum disorder [34].

A large number of NDDs are caused by loss of postsynaptic density (PSD) proteins, including *SYNGAP1. SYNGAP1* is a key regulator of synaptic plasticity and learning, primarily functioning at excitatory synapses through Ras signaling inhibition [12, 35]. Importantly, *SYNGAP1* deficiency impairs LTP, the cellular mechanism underlying memory formation, underscoring its fundamental role in synaptic plasticity. *MNK1/2* and SYNGAP1 intersect in the regulation of synaptic plasticity and memory formation. *SYNGAP1* modulates synaptic signaling through RAS-MAPK/ERK. In this pathway, SYNGAP1 controls excessive synaptic signaling by suppressing Ras activity, optimizing the signal-to-noise ratio during synaptic activation. *SYNGAP1* deficiency leads to impairments in hippocampal LTP, resulting in learning disorders and memory issues [36]. In mouse models, *SYNGAP1* heterozygous mutations are known to impair spatial learning and contextual memory consolidation [37]. A study in patients with ASD identified the *MNK-SYNGAP1* axis and found strong evidence that the genetic ASD risk factor Syngap1 regulates mTORC1 signaling and protein synthesis and that the *MNK*- *SYNGAP1* axis is crucial for ASD-associated behaviors such as social interaction, learning, and memory [18]. Although classified as a synaptic protein, several lines of evidence suggest a potential role for *SYNGAP1* in the early stages of cortical neurogenesis. In one study, embryonic mice lacking the *SYNGAP1* gene were found to be negatively affected developmentally at an early stage [38]. In humans, proper *SYNGAP1* expression is essential for the development of cognitive abilities [39]. *SYNGAP1* loss-of-function variants have been shown to be causally associated with intellectual disability, severe epilepsy, ASD, and schizophrenia [40, 41].

*SYNGAP1* deficiency is linked to cognitive impairments in animal models [42, 43]. Heterozygous *SYNGAP1* knockout mice exhibit deficits in spatial learning, working memory, social memory, and contextual memory consolidation [43].

Considering the 16.52-fold increase in SYNGAP1 expression and 8.33-fold increase in MNK1 expression in children with SLD compared to controls, our study suggests a compensatory mechanism aimed at overcoming deficits in synaptic plasticity and learning abilities. Upregulation of *SYNGAP1* and *MNK1* may attempt to enhance synaptic connections and promote learning. On the other hand, *SYNGAP1* upregulation could indicate an imbalance in excitatory-inhibitory synaptic transmission, a hallmark of many neurodevelopmental disorders. In this context, the upregulation of *MNK1* and *SYNGAP1* might reflect an adaptive response to early-life stressors or environmental factors that influence neurodevelopment. Additionally, the upregulation of these genes could be a consequence of disrupted feedback mechanisms within the MAPK/ERK pathway. In normal conditions, *SYNGAP1* acts as a negative regulator of Ras signaling, ensuring balanced synaptic activity. However, in neurodevelopmental disorders, dysregulation of this feedback loop could lead to aberrant gene expression patterns, including the upregulation of *MNK1* and *SYNGAP1*. Further studies are needed to explore these alternative explanations and clarify the precise mechanisms driving the upregulation of these genes in SLD.

*SYNGAP1-AS1*, the antisense transcript of *SYNGAP1*, is a lncRNA. Due to the wide variety of possible functions of lncRNAs, they have been identified and described to be involved in numerous biological processes, including human embryonic development and neurodevelopment. *SYNGAP1-AS1* may regulate *SYNGAP1* expression and function through epigenetic or post-transcriptional mechanisms, but the literature on the function of *SYNGAP1-AS1* remains limited. However, one study suggests that *SYNGAP1-AS1* may negatively regulate *SYNGAP1* expression. Based on this finding, it can be hypothesized that the upregulation of *SYNGAP1-AS1* may serve to downregulate *SYNGAP1* expression, as increased *SYNGAP1* levels could disrupt the signal-to-noise ratio within the pathway, thereby impairing LTP [44]. Additionally, one of the main interpretations of epigenetic mechanisms is that they serve to store information in the central nervous system. According to this view, epigenetic mechanism can alter gene expression and induce functional changes in synaptic plasticity [45]. In our study, *lncSYNGAP1-AS1* expression was upregulated (approximately 26-fold) in children with SLD compared to controls. However, this finding did not reach statistical significance, which may be attributed to the small sample size or the high variability in lncRNA expression levels [46]. Another interpretation of the results is that *SYNGAP* upregulation may disrupt the signal-to-noise ratio in synapses and cause loss of function in the MAP/ERK pathway. Therefore, upregulation of *SYNGAP1-AS1* may be aimed at regulating *SYNGAP* regulation. Our results provide preliminary evidence for the involvement of *lncSYNGAP1-AS1* in SLD; therefore, they should be interpreted more detailed with further functional studies.

In our study, *MNK1* and *SYNGAP1* upregulation did not significantly differ across SLD severity levels. This may reflect the heterogeneous nature of SLD, where gene expression is influenced by individual variability, compensatory mechanisms, or environmental factors rather than severity alone. Epigenetic modifications post-transcriptional regulation could also contribute to this outcome. Alternatively, other molecular pathways may play a greater role in severe cases.

Although our study ensured homogeneity in terms of age, gender, and socio-cultural factors, it is important to acknowledge that SLD is a multifactorial condition influenced by epigenetic, cultural, and environmental factors. Consequently, the generalizability of our findings to broader and more diverse populations remains uncertain. Future research should aim to include larger and more heterogeneous cohorts and consider integrating analyses of environmental exposures and epigenetic modifications. In this study, we used easily accessible blood samples to examine the expression of neurodevelopmental genes. However, this is a limitation of the study, as blood samples may not fully reflect brain-specific gene expression. Since brain tissue-based data are not available in SLD studies, it is important to use samples more closely related to the brain in this field. In future research, the use of alternatives such as neurons derived from induced pluripotent stem cells or cerebrospinal fluid may allow a better understanding of the molecular basis of SLD. Another limitation of our study, although it provides valuable insights into the altered RNA expression of neurodevelopmental genes in children with SLD, is the lack of protein-level validation. Future studies should include protein assays such as Western blotting or ELISA to confirm whether the observed upregulation of *MNK1* and *SYNGAP1* at the transcript level translates into increased protein expression.

## Conclusion

Our findings regarding the upregulation of *MNK1*, *SYNGAP1*, and *lncSYNGAP1-AS1* in children with SLD highlight their potential as biomarkers for early diagnosis and therapeutic targets. The involvement of the MAPK/ERK pathway and SYNGAP1 in synaptic plasticity suggests that modulating these pathways could improve cognitive function in SLD, paving the way for personalized interventions, including targeted educational strategies or pharmacological treatments. However, to confirm the generalizability and robustness of these results, replication in larger, independent, and more diverse cohorts is essential. Such efforts will strengthen the translational relevance of our findings and help advance the development of targeted diagnostic and therapeutic strategies.

## Data Availability

No datasets were generated or analysed during the current study.
