# Correction to: The *MNK-SYNGAP1* axis in specific learning disorder: gene expression pattern and new perspectives

**DOI:** 10.1007/s00431-025-06623-6

**Published:** 2025-12-01

**Authors:** Cansu Mercan Isik, Elif Burcu Tuzemen Bayyurt, Nil Ozbilum Sahin

**Affiliations:** 1 Department of Child and Adolescent Psychiatry, Faculty of Medicine, Cumhuriyet University, Sivas, Turkey; 2 Department of Medical Biology, Faculty of Medicine, Cumhuriyet University, 58140 Sivas, Turkey; 3 Department of Molecular Biology and Genetic, Faculty of Science, Cumhuriyet University, 58140 Sivas, Turkey

**Correction to: European Journal of Pediatrics (2025) 184:260**

10.1007/s00431-025-06089-6

In the originally published version of this article, the ethical statement was presented in an abbreviated form.

To provide full transparency in line with institutional and ethical standards, the following clarification has been added to the Materials and Methods section:

The initial ethical approval for participant recruitment and blood collection was granted by the Clinical Research Ethics Committee of Sivas Cumhuriyet University on 28 March 2023 (Decision No: 2023-03/10).

A subsequent approval from the Non-Interventional Clinical Research Ethics Committee of Sivas Cumhuriyet University on 21 December 2023 (Decision No: 2023-12/53) permitted the use of the same blood samples for the present study.

This correction does not affect the scientific content, results, or conclusions of the article.

The authors apologize for the oversight and thank the editorial team for their kind assistance in publishing this correction.

The original article has been corrected.

